# Suicidality among university students in the Eastern Mediterranean region: A systematic review

**DOI:** 10.1371/journal.pgph.0002460

**Published:** 2023-10-20

**Authors:** Hasti Fadakar, Jane Kim, Lauren C. Saunders, Mostafa M. Kamel, Mohsen Kianpoor, Arash Hoseyni Moghadam, Dianah Hayati, Noor Ramadhan, Tala Maragha, Maximilian Meyer, Kerry Jang, Reinhard M. Krausz

**Affiliations:** 1 Faculty of Medicine, Department of Psychiatry, University of British Columbia, Vancouver, BC, Canada; 2 School of Environmental and Life Sciences, University of Newcastle, Ourimbah, Australia; 3 Faculty of Medicine, Department of Psychiatry, Tanta University, Tanta, Egypt; 4 University of Basel Psychiatric Clinics, University of Basel, Basel, Switzerland; NIMHANS: National Institute of Mental Health and Neuro Sciences, INDIA

## Abstract

The prevalence of suicide attempts and suicidal ideation among university students is a global concern. Cultural values, social determinants, religion, and especially growing stress all play an important role in this. This systematic review aimed to identify potential protective and risk factors thought to be associated with suicidal ideation among students in the Eastern Mediterranean region and highlight the importance of developing an effective health care response. MEDLINE, CINAHL, Embase, PsycINFO, WHO Global Health Library, IMEMR, Web of Science Core Collections and Farsi and Arabic databases were searched for papers in English, Farsi, and Arabic. A combination of validated filters, free text keywords, and Mesh and Non-Mesh terms were used to retrieve relevant literature. A total of 2774 papers were found after the search, 257 selected for full-text review, and 72 papers included in the final review. Family and peer support play a potential protective role in the development of suicidal ideation among university students, while adverse life events, bullying, depression, anxiety, and other mental health conditions were identified as risk factors. Suicidality was likely under-reported due to stigma around social and cultural factors. Factors involving religion and culture may act as both protective and risk factors and require more in-depth investigation. The student population in the Eastern Mediterranean region face many challenges. The common theme of suicidality emerged as an indicator of an imbalance of resources and stress, which needs to be addressed proactively, given a most likely underreporting of suicidal ideation and attempts due to stigma.

## Introduction

Suicide is a serious mental health issue and public health problem that claims the lives of 703,000 people every year, worldwide [1]. It is estimated that for each suicide, there are between 10 to 30 attempts, and an exponentially greater number of individuals suffering from suicidal ideation [1]. While suicide occurs across all ages, genders, and ethnicities, its rate is especially concerning among adolescents and young adults, ranking the fourth leading cause of death among people aged 15 to 29 in 2019 [2]. In adolescence, it is the main reason for death in most countries.

A cross-national study of students from 12 countries showed that nearly a third (29%) had contemplated and 7% attempted suicide in their lifetime [3]. The increased risk of suicide among this population compared to the general is indicative of the stresses and challenges unique to students, such as academic stress, financial burdens, and increased social and familial pressure to meet academic expectations [4–6]. Students are further challenged by the sudden transition to university, competition with peers, and separation from family [7–9]. Academic stress is known to be a major contributor to the risk of suicide, that also increases reluctance to seek help and access formal treatment [10,11].

The World Health Organization (WHO) classifies the Eastern Mediterranean region to include 23 countries, most of which are classified as low-and-middle-income (LMICs) [12]. In 2019, LMICs accounted for 77% of all suicide deaths across the world [2]. However, official suicide rates among these nations are often reported as being lower than global rates [2], likely due to the widespread stigma and “illegal nature” of suicide in Islam [13–16]. Religion plays a prominent role in the social and cultural values of these countries and condemns suicide as an unforgivable sin [15,17]. In consequence, suicide statistics are likely not collected, underreported, or deliberately hidden from records [18,19].

Little is known about how university students in the Eastern Mediterranean region are impacted by suicide. A preliminary literature search revealed that most studies have been cross-sectional and only sampled from a single institution. The purpose of this study is to provide a thorough review of suicidality among the population of university students in the Eastern Mediterranean region and identify their social, cultural, and other specific challenges.

## Methods

### Search strategy and inclusion/exclusion criteria

The databases MEDLINE; EMBASE; Web of Science Core Collection; PsycINFO; IMEMR; CINAHL; and WHO Global Health Library were searched for published articles in English, French, Farsi, and Arabic. Articles were retrieved using search filters and database-specific filters and tools, a combination of search terms, MeSH and non-MeSH words and variations of the key terms “suicide”, “student”, and the Eastern Mediterranean countries (S1 Appendix). Farsi and Arabic databases and journals were also searched for relevant citations using translated keywords. Keywords were translated independently by two bilingual speakers and reviewed by an additional native speaker. For this review, an earlier review conducted in December 2021 was completed with updated evidence from a search in May 2023. The review was not registered.

All studies that reported proportions or mean scores of suicidal ideations among university students in the Eastern Mediterranean region were eligible to be included. Initially, both high school and university students were included. However, after completing the abstract screening, only studies including populations of university students were included. The country list was obtained from the WHO-identified countries in the Eastern Mediterranean region. Papers about other countries or regions that were not on the WHO report of the world were excluded, and only original peer-reviewed articles were selected for this review. Conference abstracts, letters, other reviews, commentaries, and editorials were excluded.

### Screening and data extraction

The PRISMA method was used for the selection of relevant articles. Two reviewers (HF, JK, AH, MMK, TM, DH, NR) independently screened the titles and abstracts of the studies using the inclusion and exclusion criteria. Any inconsistencies were reviewed by a third reviewer for inclusion or exclusion. Following the title and abstract screening, the full texts of the resulting studies were obtained and separately reviewed by two reviewers. Any discrepancies were resolved through a consensus discussion with all reviewers. A total of 72 studies were included in the review.

After screening, selecting, and evaluating the quality of selected studies, studies with missing data in their published reports were excluded, and data from the remaining studies were extracted in Microsoft Excel. Items for data extraction included information relevant to the publication (e.g., title, author, publication year), methods (e.g., type of the study, recruitment method, timeline, country, methodology of data collection and analysis), and the population of the study (e.g., sample size, age, gender, marital status, name of the school, place of residence, socioeconomic status). Population demographics, study methods, risk and protective factors of suicide were independently collected using the table. Risk and protective factors were defined as those that precede and are associated with a higher or lower likelihood of suicidality. Results were described both quantitatively and qualitatively to account for the high between-study heterogeneity observed, due to differences in instruments, environments, and mental health diagnoses.

### Study quality assessment

The Newcastle-Ottawa Quality Assessment Scale for both cohort and cross-sectional studies were employed to assess quality and evaluate bias in all the articles included in the review. There were no randomised controlled trials, case-control studies, qualitative studies, or case reports found appropriate for analysis in this study.

## Results

### Search results

**Fig 1:**
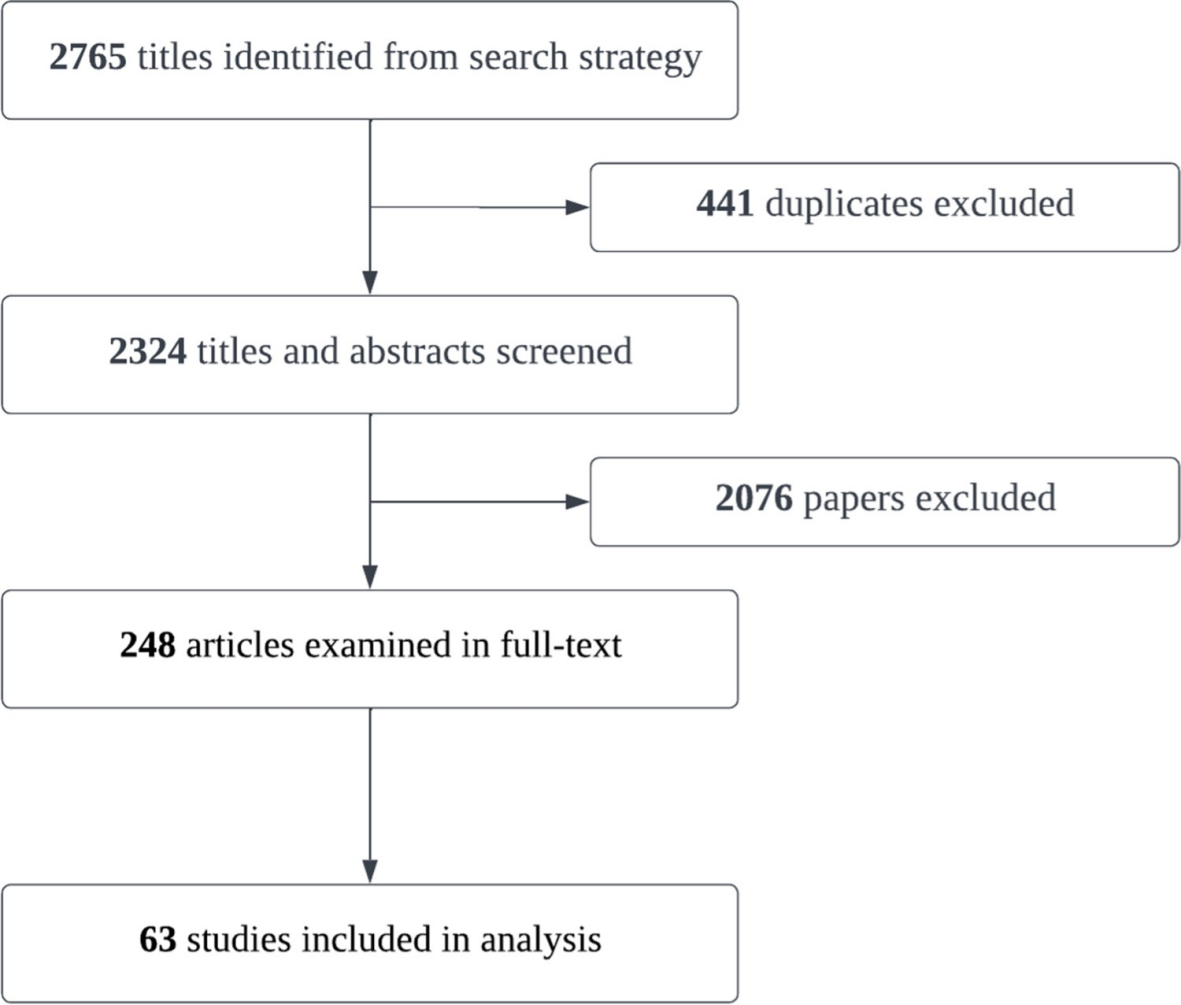
PRISMA flow diagram for study selection.

The search in MEDLINE, CINAHL, Embase, PsycINFO, WHO Global Health Library, IMEMR, Web of Science Core Collections databases as well as 3 Farsi databases including Noormags, CIVILIKA, and SID, resulted in 2774 papers that were in English and French, 2333 after the removing the duplications, of which 257 were selected for full-text review and 72 were included in the study (Fig 1). We were not able to access the full text version of any papers in Arabic as they were not available to the public.

A summary of all included studies is provided in Table 1. Students included were from twelve different countries, with the most studies conducted in Iran (N = 42), followed by Pakistan (N = 11), Saudi Arabia (N = 8), Tunisia (N = 5), and Egypt (N = 4). Other countries include Palestine (N = 3), Lebanon (N = 3), Morocco (N = 2), Kuwait (N = 2), Libya (N = 1), Sudan (N = 1), and United Arab Emirates (N = 1). A total of 42 studies disclosed the programs of students, and of those, the majority were medicine (N = 30, 71.4%). The studies’ publication years ranged from 2000 to 2016.

**Table 1:** A summary of all included studies investigating suicide in university students of the Eastern Mediterranean region.

AUTHOR, YEAR PUBLISHED	TITLE	OBJECTIVE OF STUDY	SAMPLE SIZE (N)	CITY, COUNTRY	TIMELINES	RECRUITMENT METHOD	METHODOLOGY OF STUDY AND DATA COLLECTION	METHODOLOGY OF DATA ANALYSIS	NAME OF UNIVERSITY	PROGRAM OF STUDY
Abdel-Khalek AM, et al (2002) [20]	Can personality predict suicidality? A study in two cultures.	Identify the associations and predictors of suicidality	460	Kuwait	N/A	Students within group testing sessions	Questionnaires	SPSS, factor analysis, Kaiser test	N/A	Social Science
Abdel-Khalek AM, et al (2007) [21]	Religiosity, health, and psychopathology in two cultures: Kuwait and USA.	Explore the associations among health, psychopathology, and religiosity	460	Kuwait City, Kuwait	N/A	Students within group testing sessions	Self-rating scales	t-ratios, and Pearson inter-correlations, Kaiser test, direct oblimin method	Richard Stockton College of New Jersey, University of Kuwait	Kuwait: Social Science, American: Psychology
Abdollahi A, et al (2017) [22]	Coping style as a moderator of perfectionism and suicidal ideation among undergraduate students	Explore links between adaptive & maladaptive perfectionism, emotion-focused, avoidance & task focused coping, and suicidal ideation	547	Iran	Jan-Mar 2014	Multistage sampling method	Questionnaire	Structural Equation Modeling	Islamic Azad University of Karaj	N/A
Ahmadboukani S, et al (2021) [23]	Testing Thwarted Belongingness and Perceived Burdensomeness in Suicidal Ideation and Behavior in Students: Investigating the Moderating Role of Hopelessness	Explore the interaction of hopelessness, perceived burdensomeness, and thwarted belongingness in suicidal ideation and behavior	650	Ardabil, Iran	2020–2021	Convenience sampling	Questionnaire	Pearson’s correlation and Hayes’ macro PROCESS tests.	Mohaghegh Ardabili University	N/A
Ahmadi J, et al (2014) [24]	Gender differences in depression scores of Iranian and German medical students.	Assess gender differences in depression scores of German and Iranian medical students.	200	Shiraz, Iran	N/A	Cluster random sampling	Self-reported questionnaire	Chi-square test, t-test	Shiraz University and Dusseldorf University	Medicine
Ahmadpoor J, et al (2021) [25]	Psychiatric disorders and associated risky behaviors among Iranian university students: results from the Iranian PDABs survey.	The extent of psychiatric disorders and associated factors among Iranian university students.	4261	Iran	N/A	Students during free time in campus public spaces	Questionnaire	Simple and multiple logistic regression analyses	Tehran, Shiraz, Kermanshah, Mashhad, Zahedan, Hamadan, Kurdistan, Urmia, Iran, Gilan, Birjand, Bojnord, and Rafsanjan	Medical Science
Ahmed SA, et al (2016) [26]	Forensic analysis of suicidal ideation among medical students of Egypt: A crosssectional study.	Estimate the prevalence of suicide, correlation of suicidal ideation with phases of medical studies	612	Cairo, Egypt	Mar 2016	Student participants through questionnaire	Online survey	SPSS, chi square test, logistic regression	Ainshams University and Cairo University.	Medicine
Akbari A, et al (2017) [27]	Perceived Family Functioning and Suicidal Ideation Among University Students: Hopelessness as a Moderator	Evaluate hopelessness as a moderator in the association between suicidal ideation and family functions.	337	Tehran, Iran	2014	Convenience sampling	Self-reported scales	Simple correlation and hierarchical regression by SPSS 19	N/A	N/A
Akram B, et al (2018) [28]	Coping mechanisms as predictors of suicidal ideation among the medical students of Pakistan	Explore the positive and negative coping strategies as determinants of suicidal ideation among medical students	1200	Gujrat and Lahore (Punjab Province of Pakistan)	Jan-Oct 2017	Multilevel mixed methods sampling	Questionnaire	N/A	N/A	Medicine
Almoammar S, et al (2021) [29]	Depression and suicidal ideation among dental students of Southern Saudi Arabia: A cross sectional study	Explore the association between suicidal ideation and depression during dental school	218	Saudi Arabia	Jan-Mar 2020	All dental students and interns	Questionnaire	Pearson chi-square test, one-sample binomial test, and stepwise logistic regression analysis, Shapiro-Wilk test	Khalid University college of Dentistry	Dentistry
Amiri L, et al (2013) [30]	Suicidal behavior and attitudes among medical students in the United Arab Emirates	Explore the prevalence of suicidal ideation and attempts, gender-, mood-, and religiosity-related differences	115	Al Ain, United Arab Emirates	N/A	Students of all years	Self-report survey	ANOVAs, t-test, chi-square,	United Arab Emirates University	Medicine
Amr M, et al (2013) [31]	Depression and anxiety among Saudi University students: Prevalence and correlates	Explore the prevalence of mental health issues in undergraduate college students	1696	Al-Hasa Kingdom of Saudi Arabia (KSA)	Oct-Dec 2010	A systematic random sampling technique	Questionnaire	Chi square and Z-tests, Multivariate logistic regression analysis models	King Faisal University	Agricultural Science, Education, Veterinary, Management Science, Science, Computer and Information Technology Science, Medicine, Clinical Pharmacy, Engineering and Applied Community Science.
Aryapouran S, et al (2021) [32]	Students’ Suicidal Thoughts during Covid-19 pandemic: The Role of Attachment to God, Spiritual Well-Being, and Psychological Resilience	Explore the prevalence of suicidal thoughts in students during the Covid-19 period	311	Semnan, Iran	2020	Convenience sampling	Questionnaire, self-reported scale	Pearson correlation and multivariate regression using stepwise method were used to analyze the data.	Malayer and Payam Nour University	N/A
Barrimi M, et al (2020) [33]	Les idées et les tentatives de suicide chez les étudiants en médecine au Maroc: Résultats d’une étude multicentrique = Suicidal thoughts and suicide attempts among medical students in Morocco: Results of a multicenter study	Explore the prevalence of suicidal ideation and attempts, and clarify the factors associated among medical students.	600	Oujda, Morocco	Oct 2017	Study advertised through social media for medical/pharmacy students.	Anonymous online survey	Chi2 test or Fisher’s test	Université Mohamed	Medicine & Pharmacy
Bibi A, et al (2021) [34]	Mental health, suicidal ideation, and experience of bullying among university students in Pakistan	Explore the problems of poor mental health, lack of treatment, and suicidality, prevalence of history of bullying in university students.	355	Pakistan	N/A	Study advertised through social media	Anonymous online survey, questionnaire	t-tests and chi-square test	N/A	N/A
Borji M, et al (2019) [35]	Prediction of Suicidal Ideations Based on Meaning in Life and Early Maladaptive Schemas among University Students	Explore the predictive role of early maladaptive schemas and meaning in life in suicidal ideation among university students.	240	Tehran, Iran	2016–2017	Multistage cluster sampling method	Questionnaire, self-reported scale	SPSS-23 Software using Pearson correlation coefficient and regression analysis.	Iran University of Medical Sciences	Medical Science
Dadfar M, et al (2021) [36]	The patient health questionnaire-9 (phq-9) as a brief screening tool for depression: A study of iranian college students	Assess the psychometric properties of PHQ-9 and severity of depression symptoms in Iranian university students	260	Tehran, Iran	N/A	Volunteer convenience sample	N/A	Pearson correlation coefficients and Principle Components Analysis (PCA) with a varimax rotation	Iran University of Medical Sciences	Medicine
Dar S, et al (2022) [37]	Psychosocial factors and suicidal ideation in medical students	Investigate the relationship between psychosocial factors (depression, anxiety, stress sand social support) and suicidal ideation in medical students	100	Lahore, Pakistan	Feb-Nov 2016	Student participants through questionnaire	Questionnaire	Hierarchical multiple regression analysis	Services Institute of Medical Sciences College and King Edward Medical University	Medicine
Elhadi M, et al (2020) [38]	Psychological Impact of the Civil War and COVID-19 on Libyan Medical Students: A Cross-Sectional Study	Explore the psychological status of Libyan medical students throughout the COVID-19 pandemic and civil war	2430	Tripoli, Al- Zawia, Misrata, Sebha, Gharyan, Albayda, Benghazi, Al Khums, Tarhuna, Alzintan, Tobruk, and Sabratha. Libya	Apr-May 2020	Voluntary online recruitment of medical students	Anonymous surveys	Independent-samples t-test, chi-square test, Spearman’s rank order correlation	15 medical schools and colleges in Libya	Medicine
Eskin M, et al (2020) [39]	Associations of religiosity, attitudes towards suicide and religious coping with suicidal ideation and suicide attempts in 11 Muslim countries	Explore the associations of religiosity & suicide acceptance with suicidality	7427	Iran, Jordan, Lebanon, Pakistan, Palestine, Saudi Arabia, Tunisia, Egypt (also Turkey and Azerbaijan)	2016	Convenience sample by local researchers	Self-administered questionnaires	F and χ2 tests, Poisson distribution, binomial distribution	N/A	N/A
Eskin M, et al (2021) [40]	Cultural and interpersonal risk factors for suicide ideation and suicide attempts among Muslim college students from 11 nations	Identify the links between cultural factors and interpersonal elements with suicidal ideation and attempts	5115	Iran, Jordan, Lebanon, Pakistan, Saudi Arabia, Tunisia, Egypt (Azerbaijan, Indonesia, Turkey, Palestine)	N/A	Convenience sampling	Questionnaire	Confirmatory Factor Analysis (CFA) modelling	N/A	N/A
Fekih-Romdhane F, et al (2020) [41]	Is Religiosity Related to Suicidal Ideation Among Tunisian Muslim Youth After the January 14th Revolution?	Assess the relationship between religiosity and suicide thoughts in university students	303	Tunis, Tunisia	Feb-May 2017	N/A	Semi-structured interview form	Mann–Whitney test, Kruskal–Wallis test, Spearman’s ranking correlation matrix, Multiple linear regression calculations	Faculty of Medicine of Tunis, University of Human and Social Sciences of Tunis, National Engineering School of Tunis	Medicine, Human and Social Sciences, Engineering
Fekih-Romdhane F, et al (2021) [42]	The role of personal factors and learning environment in suicidal ideation among Tunisian medical students	Explore the prevalence of suicidal thoughts among medical students in Tunisia	390	Tunis, Tunisia	N/A	Students currently enrolled in the second cycle of medical studies	Anonymous questionnaire	SPSS, Mann–Whitney test, r Kruskal–Wallis test, multivariate hierarchical regression	N/A	Medicine
Fekih-Romdhane F, et al (2021) [43]	Suicidal ideation, suicide literacy and stigma, disclosure expectations and attitudes toward help-seeking among university students: The impact of schizotypal personality traits	Assess suicidal ideation and determine factors that may have associations with help-seeking attitudes in high schizotypal individuals	504	Tunis, Tunisia	Sep-Dec 2020	Students enrolled in 3 major universities in Tunis during the 2020–2021 academic year	Questionnaire	Hierarchical multiple regression modelling	Institute of Advanced Business Studies, Faculty of Human and Social Sciences of Tunis, and Preparatory Institute for Scientific and Technical Studies	Business Studies, Human and Social Sciences
Ghadampour E, et al (2017) [44]	Relationships among Cyberbullying, psychological vulnerability and suicidal thoughts in female and male students	Explore the association between cyberbullying, psychological vulnerability & suicidal ideation in university students	120	Khorramabad, Iran	2016–2017	Random stratified sampling method	Questionnaire	Pearson correlation and Fisher Z with Assistance SPSS-20 software	N/A	N/A
Ghaderi D, et al (2018) [45]	The comparison of profile and personality traits in the female students with and without suicidal thoughts	Compare personality traits & profile in students with and without suicidal ideation	396	Urmia, Iran	2017	Multistage cluster sampling	Self-reported scale	Data was analyzed using MANOVA by SPSS software	Isfahan University of Medical Sciences	Medical Science
Goldney R, et al (1998) [46]	Suicidal ideation in Sudanese women	Assess suicidal thoughts among women in Jebel Aulia displaced-persons area and female students of Ahfad University for Women	29	Omdurman, Sudan	N/A	Psychology students, displaced persons attending a clinic or simply on availability	Questionnaire	N/A	Ahfad University	Psychology
Habib, O et al (2022) [47]	Prevalence of Suicidal Ideation and Planning in Senior Medical Students	To assess the prevalence rates of suicidal ideation and planning in medical students	248	Lahore, Pakistan	Jun-July 2022	Voluntary self-administered questionnaire in fourth year and final year medical students	Questionnaire	Descriptive statistics	Private medical college	Medicine
Habibi F, et al (2021) [48]	Frequency of Thoughts and Planning for Suicide Attempt in Paramedical Students of Rafsanjan University of Medical Sciences in 2019: A Cross- Sectional Study	Explore the frequency of suicidal thoughts and suicide planning among paramedical students	209	Rafsanjan, Iran	N/A	Census sampling	Questionnaire	Chi square, one- way ANOVA and logistic regression	Rafsanjan University of Medical Sciences	Paramedicine
Hakami R (2018) [49]	Prevalence of Psychological Distress Among Undergraduate Students at Jazan University: A Cross-Sectional Study.	Explore the prevalence of psychological distress in Jazan undergraduate students	450	Jazan, Saudi Arabia	Mar 2017	Stratified sampling and random sampling	Self-reported questionnaire	Independent sample t-tests	Jazan University	Business Administration (34.9%), Sciences (22.2%), Computer Sciences (19.6%), Applied Medicine (8.0%), Sciences (22.2%), Pharmacy (5.3%)
Hamdan S, et al (2021) [50]	Prolonged exposure to violence: Psychiatric symptoms and suicide risk among college students in the Palestinian territory	Explore the association of PTSS, sleep problems, and depressive symptoms with suicidal ideation	303	West Bank of Palestine	N/A	Snowball sampling	Questionnaire	T tests or chi-square tests, hierarchical logistic regression models, exploratory path analysis model	N/A	N/A
Heydari A, et al (2014) [51]	Development of suicidality within socioeconomic context: Mediation effect of parental control and anomie	Explore the impact of feeling of anomie and perceived parental control in the process of suicidality development	350	Ahvaz, Iran	N/A	Randomly chosen	Questionnaires	Correlations and coefficients	Shahid Chamran University	N/A
Hidarisharaf P, et al (2015) [52]	Relationship of quality of life, spirituality and resilience with suicidal thoughts in students	Determine the association between the quality of life, resilience and spirituality with suicidal thoughts among university students	200	Kermanshah, Iran	N/A	Cluster sampling	Questionnaire	Statistical analysis	Kermanshah Razi Faculty of Social Sciences	N/A
Inam S.B (2007) [53]	Anxiety and Depression among Students of a Medical College in Saudi Arabia.	Explore the prevalence of depression and anxiety in medical students.	302	Buryadeh, Saudi Arabia	N/A	Voluntary self-administered questionnaire	Depression scale	Epi info version 6.0 tables, chi square test	College of Medicine, Al-Qaseem University	Medicine & Paramedicine
Khaleghi M, et al (2021) [54]	Emotional Schemas Contribute to Suicide Behavior and Self-Harm: Toward Finding Suicidal Emotional Schemas (SESs)	Identify the risk factors for suicide behaviour, and ideation, and self-harm, among university students.	375	Karaj, Iran	N/A	Students from Khazami University campus	Self-rating scales	Correlation and logistic regression	Kharazmi University, Karaj campus, Iran	N/A
Khokher S, et al (2005) [55]	Suicidal Ideation in Pakistani College Students	Explore the prevalence of suicidal ideation and examine its relationship with sociodemographic factors.	217	Karachi, Pakistan	2005*	Medical student body enrolled in the M.B–B.S degree program	Questionnaires	SPSS	Private medical college in Karachi	Medicine
Khosravi M, et al (2020) [56]	The relationship between neuroticism and suicidal thoughts among medical students: Moderating role of attachment styles.	Discover the relationship between neuroticism, suicidal ideation, and other psychopathological variables involved.	376	3 major cities Iran	N/A	Multistage sampling technique	Questionnaire	t-test, one-way ANOVA variance method, Cramers V test, Pearson correlation coefficient, SPSS	N/A	Medicine
Kiaei Y, et al (2022) [57]	The Relationship Between Suicidal Ideation and Perfectionism in Iranian Students: The Mediating Role of Self-criticism	Investigate how self-criticism mediates the relationship between suicidal ideation and perfectionism	300	Tehran, Iran	2008–2019	Convenience sampling	Questionnaire	Structural equation modelling	University of Science and Culture, Tehran, Iran	N/A
Kilbert J, et al (2021) [58]	A Protective Model for Suicidal Behaviors in American and Pakistani College Students.	Explore the effects of depressive symptoms and life-enhancing activities on suicidal behaviors.	449	Karachi, Pakistan	N/A	Undergraduate students enrolled in psychology courses at selected universities	Survey data (scales, questionnaires)	linear regressions, SPSS	N/A	Grade A level to graduation educational institutions
Landrault H, et al (2020) [59]	Suicidal ideation in elite schools: A test of the interpersonal theory and the escape theory of suicide	Explore the power of interpersonal and escape theories of suicide in prediction of suicidal ideation.	306	Marrakech, Morocco	N/A	Students attending preparatory classes	Online Questionnaire	Pearson Bivariate correlations, multiple regression analysis	Private medical school of university in the Marrakech-Safiregion	Medicine
Madadin M, et al (2021) [60]	Suicidal ideation among medical students in Dammam, Saudi Arabia: A cross-sectional study	Appraise suicidal ideation and its associating factors among medical students.	265	Dammam, Saudi Arabia	N/A	Medical students enrolled at university	Questionnaire	SPSS, chi-square tests and logistic regression analysis	Imam Abdulrahman Bin Faisal University	Medicine
Modaresesabzevari S, et al (2016) [61]	The role of Rumination Subtypes, Hopelessness and Depression in the Prediction of Suicidal Ideations in College Students	Identify the predictive role of rumination and its subtypes along with hopelessness and depression in suicidal thoughts	177	N/A	N/A	Convenience sampling method	Questionnaire	N/A	N/A	N/A
Mohamed MY, et al (2023) [62]	Depression and suicidal ideations in relation to occupational stress in a sample of Egyptian medical residents	Determine the rate of depression and severity in a group of Egyptian residents	220	Egypt	Mar 2019-Sep 2020	All residents with no diagnosed major medical illness or antidepressant use for more than 2 weeks before residency	Interview	Descriptive statistics	Ain Shams University	Internal Medicine, Surgery, Obstetrics and gynecology, Pediatrics, Anesthesia, Others
Mohammadinia N, et al (2012) [63]	Assessing suicidal ideation frequency in medical students	Explore the frequency of suicidal thoughts and its associating factors in university students.	288	Iranshahr, Iran	2010	All students	Questionnaire and self-reported scale	Chi-square test, Fisher and Pearson correlation in SPSS16.	Zahedan University of medical sciences	Nursing, Midwifery & Medical Emergency
Monirpoor N, et al (2014) [64]	Vulnerability to substance abuse and the risk of suicide in students of region 12 of Islamic Azad university.	Assess the vulnerability of university students to substance use and suicide risk in students.	1053	Iran	N/A	Stratified sampling	Questionnaire	Independent groups t-test, one-way variance analysis	Islamic Azad University	N/A
Mousavi SG, et al (2012) [65]	Suicidal ideation, depression, and aggression among students of three universities of Isfahan, Iran in 2008.	Assess the prevalence of aggression, suicidal thoughts, and depression in university students.	435	Isfahan, Iran	Feb-Sep 2008	Students present in public placed of universities	Questionnaire	t-test, analysis of variance (ANOVA), Mann-Whitney H and Kruskal-Wallis U tests were used for BDI and BSSI scores, Pearson’s correlation test	Isfahan University, Isfahan University of Medical Sciences, and San’ati University of Isfahan.	Medicine, Economics, Laws, Education, Biology, Language and other sciences, Engineering and other industrial sciences
Mousavi SG, et al (2005) [66]	Relative frequency of suicidal ideation in students of Isfahan universities in 2005	Explore the relative frequency of suicidal thoughts among university students.	300	Isfahan, Iran	2005	Random sampling	Questionnaire	Chi square test was used for data analysis using SPSS software.	Isfahan Universities	N/A
Movahedi Y, et al (2013) [67]	Predicting students’ suicidal tendencies based on religiosity, social support, family environment and depression	Explore the predictive value of family atmosphere, religiosity, depression and social support for students’ suicidal ideation.	1067	Khorramabad, Iran	2011–2012	Multistage cluster sampling method	Questionnaire	Correlation and regression analysis	Lorestan University of Medical Sciences	Medical Sciences
Mufti RE, et al (2022) [68]	Psychological well-being and its effect on suicidal behavior among medical students in Madinah	Measure the psychological well-being of students and assess its impact on the development of suicidal behavior	308	Al-Madinah, Saudi Arabia	Jan-Feb 2022	Online sampling method	Questionnaire	SPSS, ANOVA tests and descriptive statistics	Al-Rayan colleges and Taibah University	Medicine
Muneeb NUA, et al (2022) [69]	Psychological strain and suicidal ideation in young university students during Covid-19 outbreak: the mediating roles of rumination and depression	Examine the relationship between psychological strain, suicidal ideation, rumination, and depression	400	Pakistan	N/A	Convenience sampling	Online Questionnaire	Structural equation modelling	Universities of Rawalpindi or Islamabad	Bachelor’s, Master’s, or Ph.D. Programs
Naghavi A, et al (2020) [70]	Accurate Diagnosis of Suicide Ideation/Behavior Using Robust Ensemble Machine Learning: A University Student Population in the Middle East and North Africa (MENA) Region	Detect suicide behaviour and ideation through a machine learning approach.	511	Isfahan, Iran	Mar-May 2020	Online sampling method	Anonymous questionnaire	ensemble learning Mann-Whitney U Test	University of Isfahan	Liberal Arts (60.7%), Basic Sciences (5.8%), Engineering Sciences (23.6%), and Medical Sciences (7.2%)
Naseem S, et al (2017) [71]	Suicidal Ideation, Depression, Anxiety, Stress, And Life Satisfaction Of Medical, Engineering, And Social Sciences Students.	Explore the difference in the level of suicidal ideation, depression, anxiety, stress, and life satisfaction in students of different programs.	300	Karachi, Pakistan	N/A	Purposive sampling	Questionnaires	ANOVA with Tukey’s HSD, t-test	31 universities, 7 public 24 private,	Medical, Engineering, and Social Sciences
Nemati Sogolitappeh F (2013) [72]	Predicting students’ suicidal ideation based on depression, borderline personality disorder, religiosity, social support and coping strategies	Explore the role of borderline personality disorder, social support, religiosity, depression and coping strategies in predicting suicidal thoughts in students.	361	Tabriz, Iran	2014	Multistage cluster sampling	Self-reported scales and Questionnaire	Pearson correlation coefficient and multiple regression analysis.	Tabriz University	Human Science (35.73%), Basic Science (41.27%), Technology (22.99%)
Panaghi L, et al (2010) [73]	Suicide Trend in University Students during 2003 to 2008	Investigate associated factors of suicide in university students.	337	Iran	2003–2008	Suicide data of students collected during one retrospective and one prospective stage through student profiles and family member interviews	Assessment of student profiles and family interviews	SPSS 14	All Universities in Iran	Human Science (40.5%), Engineering (26.2%), Medicine & Pharmacy (7.1%), Nursing & Paramedicine (4.8%), Accounting & Management (19%), Basic Science (2.4%),
Poorolajal J, et al (2019) [74]	The top six risky behaviors among Iranian university students: a national survey.	Explore the prevalence of several risk-taking behaviors such as illicit drug use and suicidal attempts among Iranian university students.	4261	Iran	N/A	Students during free time in campus public spaces	Questionnaire	Simple and multiple logistic regression analyses	Tehran, Shiraz, Kermanshah, Mashhad, Zahedan, Hamadan, Kurdistan, Urmia, Iran, Gilan, Birjand, Bojnord, and Rafsanjan	Medical Science
Poorolajal J, et al (2017) [75]	The Prevalence of Psychiatric Distress and Associated Risk Factors among College Students Using GHQ-28 Questionnaire	Explore the prevalence of psychiatric distress and its associated factors in students.	1259	Hamadan, Iran	Jan-May 2016	Proportional random sampling and random sampling	Questionnaire	Chi-squared test, simple and multiple logistic regression analysis	Hamadan University of Medical Sciences	Medical Science
Pournaghash-Tehrani SS, et al (2021) [76]	The Impact of Relational Adverse Childhood Experiences on Suicide Outcomes During Early and Young Adulthood	Identify the impact of adverse childhood experiences on suicidal outcomes of Iranian students.	487	Iran	Apr-May 2018	Multistage sampling method	Questionnaire	Descriptive statistics, Fisher’s exact test, chi-square test, a series of univariate binary logistic regression	University of Tehran	Humanities (52.2%), Engineering (25.5%), Basic Sciences (22.4%).
Poursharifi H, et al (2012) [77]	The Role of Depression, Stress, Happiness and Social Support in Identifying Suicidal Thoughts in Students	Explore the predictive value of psychological constructs including stressfulness of life, happiness, depression and social support on the rate of students’ suicidal ideation.	1094	Tehran, Iran	N/A	N/A	Self-reported scales	Multiple regression techniques	Tehran University	N/A
Raeisei A, et al (2015) [78]	The relationship between personality styles of sociotropy and autonomy and suicidal tendency in medical students.	Explore the relationship between personality styles and suicidal intention in medical students.	102	Zahedan, Iran	2002–2003	All medical students who passed internship wards	Questionnaires	Inferential statistical analysis (descriptive and correlational)	Zahedan University of Medical Sciences	Internship ward
Rahimi N, et al (2017) [79]	The relationship between spiritual health and the incidence of suicidal ideation in nursing and midwifery students of Rafsanjan University of Medical Sciences in 2014	Explore the association between spiritual welfare and the outbreak of suicidal thoughts in students.	233	Rafsanjan, Iran	2015	Census method	Questionnaire	SPSS- version 20 and parametric statistical tests.	Rafsanjan University of Medical Sciences	Nursing and Midwifery
Rashid S, et al (2017) [80]	The relationship between Death Anxiety, Mattering, Perceived Burdensomeness and Thwarted Belongingness with Suicidal Behavior in college students (The Interpersonal-Psychological Theory for suicide)	Explore the association of suicidal behavior with death anxiety, perceived burdensomeness, mattering and thwarted belongingness in university students.	331	Ardabil, Iran	N/A	Convenience sampling	Questionnaire	descriptive statistics indices, Pearson correlation analysis, and multivariate regression by using SPSS ver. 22.	The University of Mohaghegh Ardabili	N/A
Rashid S, et al (2016) [81]	The relationship between perceived social support, perceived burdensomeness and thwarted belongingness with suicidal behavior in college students (the interpersonal-psychological theory for suicide)	Explore the association between thwarted belongingness, perceived burdensomeness and perceived social support with suicidal behavior in university students.	295	Ardabil, Iran	N/A	Convenience sampling	Questionnaire	Pearson correlation analysis and multivariable regression	Mohaghegh Ardabili University	N/A
Sadri Damirchi E, et al (2018) [82]	The Relationship between Sense of Coherence and Cognitive Emotion Regulation with Suicidal Thoughts among Students at University of Mohaghegh Ardabili in 2017	Explore the relationship between cognitive emotion regulation and sense of coherence with suicidal thoughts in students.	367	Ardabil, Iran	2017	Cluster sampling method	Questionnaire	Descriptive statistics, multiple linear regression	University of Mohaghegh Ardabili	Psychology (27.5%), Human Sciences (14.6%), Basic Sciences (16.8%), Technology (10.9%), Agriculture (16.5%), Mathematics (13.7%),
Sadri Damirchi E, et al (2019) [83]	The Role of Cognitive Flexibility and Perceived Social Support in Predicting Suicidal Tendency in University Students	Identify the role of perceived social support and cognitive flexibility in predicting suicidal tendency among medical students.	220	Ardabil, Iran	2016–2017	Convenience sampling	Questionnaire	Pearson correlation coefficient and regression analysis	N/A	N/A
Salman M, et al (2022) [84]	Self-harm and suicidal ideation in Pakistani youth amid COVID-19 pandemic: findings of a large, cross-sectional study	Assess self-harm and suicidal ideation among university students	1134	Pakistan	N/A	Convenience sampling	Online survey	Independent t-tests and chi-square tests	University of Lahore, University of Punjab, Gulab Devi Educational Complex and University of Veterinary and Animal Sciences	Health Sciences, Non-Health Sciences
Shahbaziyankhonig A, et al (2020) [85]	Discriminative Role of Moral intelligence, Mindfulness and Cell Phone Addiction in Suicide Probability among Students	Differentiate students with high and low suicide probability based on mindfulness, moral intelligence and cell phone addiction.	355	Tabriz, Iran	2018–2019	Multistage cluster sampling method	Questionnaire, self-reported scale	Statistical test discriminant analysis	N/A	Agriculture (11.6%), Psychology (15.2%), Economics (17.2%), Technology (9.3%), Sciences (14.2%), Literature (12.6%), Law (19.9%)
Shamsaei F, et al (2019) [86]	Relationship between stress, depression, anxiety and suicide ideation in nursing students: A cross-sectional study	Explore the relationship between depression, stress, anxiety and suicidal thoughts in nursing students.	342	Iran	Oct-Dec 2017	Selected by Census sampling	Self-reported Questionnaire	Pearson’s correlation, multiple regression analysis	Hamadan University	Undergraduate
Talih F, et al (2018) [87]	Examining burnout, depression, and attitudes regarding drug use among Lebanese medical students during the 4 years of medical school	Assess the prevalence of anxiety symptoms, depressive symptoms and burnout and evaluate attitude toward substance use in medical students.	176	Beirut, Lebanon	Sep-Dec 2016	All medical students at any stage of medical education	Anonymous online survey	Pearson’s chi-squared or Fisher’s exact test, ANOVA with Bonferroni correction, multivariate logistic regression.	American University of Beirut Faculty of Medicine	Medicine
Tarsafi M, et al (2015) [88]	The defeat-entrapment theory versus Beck’s hopelessness theory of depression and suicidality: A cross-national analysis in Iran and the United States	Evaluate entrapment, hopelessness, defeat and depression measures in American and Iranian students.	340	Iran	N/A	Convenience samples	Questionnaires	Pearson correlations, factor analysis, and multiple regressions	N/A	Psychology (US), Counselling (Iran), Undergraduate programs
Valikhani A, et al (2015) [89]	Forecasting suicidal thoughts based on components of borderline and schizotypal personality in medical students	Determine whether schizotypal and borderline personalities are associated with suicidal ideation in students.	250	Shiraz, Iran	N/A	Convenience sampling	Questionnaire	SPSS	Shiraz University of Medical Sciences	N/A
Zarei M (2021) [90]	The relationship between resilience and suicidal ideation among university students in Tehran	Explore the relationship between resilience and suicidal thoughts among university students.	397	Tehran, Iran	2018–2019	Multistage cluster sampling	Questionnaire	SPSS	Public Universities in Tehran	N/A
Zemestani M, et al (2023) [91]	Associations between Sleep Disturbance and Suicidal Ideation Severity in Iranian University Students: Evaluating Emotion Regulation Difficulties and Distress Tolerance	Evaluate whether emotion regulation difficulties and distress tolerance explain the association between sleep disturbance and suicidal ideation in Iranian university students	679	Kurdistan region, Iran	Feb-Apr 2021	Convenience sampling	Online survey	Structural equation modelling	University students in Iran	N/A

#### Risk factors

The risk factors identified in this review include demographic and personal factors, mental health factors, and school-related factors. The demographic factors that increase the likelihood of suicidal ideation in Eastern Mediterranean students include being single [64,92], being part of the LGBTQI+ community [75], being widowed or otherwise separated from a partner [56], and residing in, or having connection to, high politicised regions [46]. Living circumstances, specifically living alone, also increased the likelihood of having suicidal thoughts compared to students living with roommates [56].

Only 4 studies found higher rates of suicidality in females than males [26,56,63]; the remainder showed no significant differences [24,29,48,49,55,60,61,64,65,93]. Two studies found males to be associated with greater suicidal behaviour [28,31]. Specific to females, the overriding expectation that familial duties should take precedence over their education or other personal goals has been positively associated with increased suicidal behaviour [28,94]. However, most studies also reported having a smaller sample of males in comparison to females [21,22,29,34,38,45,70,94].

Personal risk factors include adverse life events, negative family environments, and substance use. There is a non-linear association between negative life events and suicidal ideation [40]. Factors including witnessing physical violence, familial mistreatment or neglect, and sexual abuse significantly enhance the risk for present suicidality, while parental separation and neglect significantly increase future risk for suicidal ideation [76]. The feeling of helplessness within in the family, or perceived parental control has been observed to have significant, positive relationship with suicidal ideation [51]. In one study of medical students, 10% reported that the decision to study medicine was imposed on them by family, resulting in their increased suicidality [42]. Consumption of psychoactive substances, in particular illicit drugs, has also been associated with suicidal ideation [26,33].

Mental health symptoms are among the most consistently reported risk factors for suicide. Depression is consistently highly correlated with suicidal ideation in students [34,41,53,61,65,67,70–72,77,86,94,95], to an extent that it has been observed to have the strongest predictive power over suicidality [72,77]. Suicidal ideation was found to be increase by the severity of depression [74,75]. Females with suicidal ideation were more likely to experience depressive symptoms than their male counterparts [49,53]. Frequently, depression occurred with other mental health conditions, including but not limited to; anxiety, stress, panic, and pessimism [21,24,38,53,70,71], which compounds the likelihood of suicidal ideation [38]. Anxiety was also present in a large number of students with suicidal ideation [21,31,33,34,38,42,53,70,71,86]. Similar to depression, females had a higher prevalence of anxiety than males [49,53]. History of taking anxiety medication was also found to be significantly associated with suicidal ideation in students [79]. Other symptoms including neuroticism, borderline and schizotypal personality disorders, perfectionism, and conditions affecting sleep quality, were also associated with enhanced suicidality in the Middle Eastern student population [42,56,87,89].

Several school-related risk factors were associated with student suicide. While the majority of papers did not address the impact of bullying on suicidal ideation, those that did found that an overwhelming percentage of students had been bullied at least once throughout their lifetime [34], which significantly increased the likelihood of suicidal ideation [34,76]. While both genders reported high rates of bullying, males had higher risk of suicide from school events in comparison to females [76]. Feelings of anomie were observed to have a significant, positive correlation to suicidality [35,51,76]. Females’ suicide outcomes were affected more by feelings of anomie compared to males [76]. There were considerable inconsistencies between studies investigating university programs and their correlation with suicidal ideation. Some papers posited that program choice may affect risk of suicidal ideation [70,76], whereas others argue that there is no significant relationship [63,71]. Most studies agree that studying medical sciences are a particularly high-risk group [26,28,31,60,70]. Level of education, specifically early-year and bachelor level, was also significantly correlated to suicidal ideation [26,63,70]. Generally, heavy academic workloads, academic pressure, fear of failure, and lengthy courses all contributed to an increased risk of suicidal ideation in students [28,59]. There is evidence that just being enrolled in university classes is associated with suicidal ideation [33]. The societal pressure surrounding education and an expectation to perform, in some circumstances regardless of poor educational policies or less than ideal learning environments, is a major contributor to suicidality among students [34,42].

#### Protective factors

Interpersonal factors such as family and social support networks were identified as protective factors. Familial support is reported to be the primary source of support for students [26], seemingly acting as a mitigating factor against suicidal ideation [26,31,72]. Perceived support from family has been observed to have an inverse relationship with the risk of suicidal ideation [26,27,31,67,72,83]. Particularly, living with family members and effective parental communication were found to be especially protective [26,30,31,70]. Outside of family members, multiple studies also observed a strong, negative correlation between suicidality and peer support [41,67,80]. Only one study found no significant association between social support and suicidal ideation [37].

#### Both risk and protective factors

Sociocultural and religious factors were found to be both positively and negatively associated with suicide. Middle Eastern nations overwhelmingly favour traditional beliefs and conservative social values [34,49,94]. This includes, but is not limited to, the stigmatisation of aberrant mental health and the strong condemnation of suicide, to the extent of prohibition in some localities [49,53]. Consequentially, these social norms have directly contributed to both a lack of mental health professionals and an underutilisation of services that do exist, and high rates of psychological distress in the student population [34,60,94]. While the stigmatisation of suicide has not been observed to be directly correlated with suicide in any of the studies, it has a number of indirect, negative impacts on Middle Eastern students, such as the avoidance of mental health services [34,53]. Medical students in particular showed strong disapproval towards suicide from the belief in punishment in the afterlife post-suicide [30]. One study has directly attributed suicidal stigma with a lower prevalence of suicidal thoughts reported in their sample population [49]. This also raises issues concerning the accuracy of reporting, with the negative stigma potentially creating an inhibitory effect and skewing official suicide statistics [30,53].

Suicidal ideation was found to be negatively associated with religiosity [21,39,41,67,72,79,92]. One study noted negative religious coping being significantly associated with suicide attempts, possibly due to the gradual loss of religious commitment when faced with adverse life experiences [39]. However, there was no significant association between negative religious coping and suicidal ideation [39]. Two studies also reported a negative association between religiosity and the acceptance of suicide [30,39]. In some studies, positive religious coping and religion were considered protective factors against suicidal ideation [39,72]. Students who expressed stronger religious beliefs were more likely to view suicide as an indicator of mental illness and demonstrated greater tendency and preparedness to support a suicidal friend [30]. One study found no significant association between attitudes towards suicide and religiosity or the knowledge of the Islamic prohibition of suicide [39]. Lastly, some studies indicated that suicidality might be under-reported in Eastern-Mediterranean countries due to religious restrictions [46] and suicide being considered a social taboo [34,60].

### Study quality assessment

The 72 papers included in the review were all cross-sectional studies with a single exception. The lone paper was a cohort study and deemed of “good” methodological quality by the Newcastle-Ottawa Quality Assessment Scale, with a score of 8 of a possible 9, only losing a mark on the ability to compare cohorts based on the study design criteria. The overwhelming majority of the cross-sectional studies were rated as “good” methodological quality (a score of 6 or above up to 10). Eight papers were of “moderate” quality (with a score of 5) with flaws in their representativeness of the sample, affirming the exposure of the sample, or having an insufficient sample size. One paper was judged to be of “poor” methodological quality with a score of 3, with major issues regarding sample size, comparability, and assessment of the outcomes, amongst others (see S1 Table). Lastly, 67 papers discussed suicidal ideation, 4 discussed suicide attempts, and 5 discussed both, and none of the papers focused on fatalities through suicide.

## Discussion

To our knowledge, this is the first study to comprehensively explore and review literature around students’ suicide in the Eastern Mediterranean region. Our goal was to provide a summary of the available studies around this population to guide and inform prevention and intervention practices.

Our review revealed that adverse life events, bullying, depression, anxiety, and other mental health conditions were identified as risk factors of suicidal ideation in university students, whereas family and peer support can play a protective role. Religion and cultural factors may act as both risk and protective factors. Sociocultural factors play a crucial role in developing a sense of belonging that protects individuals from feeling alienated and outcasted from the community. However, extreme emphasis on these norms and traditions may contrarily have a destructive effect on the mental well-being of people within the community.

The presented studies confirm that religious affiliation generally protects individuals against suicidal ideation, consistent with previous findings in which religiosity was shown to be significantly and negatively correlated with suicidal ideation [21]. However, the prohibition of suicide by many religions such as Islam may indirectly perpetuate the stigma around suicide. More religious individuals are more likely to consider suicide an unforgivable sin, intertwining with stigmatizing attitudes towards suicide. As a result, those experiencing suicidal thoughts may refuse to seek help due to the fear of being stigmatized and perceived as sinful. Moreover, the condemned view of suicide within religion may result in the under-reporting of suicide or attempting to disguise it, leading to inaccurate suicide statistics, and deflected attention from this concerning issue.

Students in the Eastern Mediterranean region are a high-risk population, not only due to their educational challenges, but from additional stressors specific to this region such as accessibility, national security concerns, social pressure, cultural restrictions, and potential lack of academic freedom. While all students require greater support, those under higher academic strain in the fields of humanities, sciences, or medicine may require focused interventions to reduce their perceived stress.

Furthermore, there is a substantial disparity between individuals needing care and those with access to mental health services within this region. The case is even worse for students due to the lack of awareness, support, and resources available to them. In a sample of Egyptian medical school students, Kamel et al. stated that only 6% of students with mental health problems could access professional assistance [25]. This is likely to exacerbate mental health conditions for students, which are predisposing factors for the development of suicidal ideation and behaviour.

The unique sociocultural fabric of the Middle Eastern youth and adolescents must be considered in order to develop safe, accessible, and trust-worthy resources that address their barriers to service utilization. Due to their significant role in the decision making of youth, family and peer support should be highly integrated in the new system. The heavy use of internet within the population of university students and their familiarity with this technology could be leveraged to better guide youth through the mental healthcare system with web-based solutions dedicated to psychoeducation and counselling services that fit students’ needs [20,23,96]. As a good start, looking at the Egyptian experience, the ministry of health in collaboration with the WHO and experts from the university of British Columbia, has just launched a platform for E-Mental Health, as an innovative and appropriate alternative, considering the current inadequacy in the current youth mental health care system with high demand from youth to use this platform [36].

This study has some limitations. Firstly, the chosen countries for this study were based on the WHO list of Eastern Mediterranean countries which does not include all the countries commonly considered as the Middle East (e.g., Turkey and Algeria). Secondly, we were not able to access Arabic and Hebrew databases. Hence, papers written in these languages were not included. Thirdly, literature representing some Eastern Mediterranean countries is scarce. Therefore, some countries and their populations were not represented (e.g., Afghanistan), whereas other countries were overrepresented (e.g., Iran). Additionally, Israel and Palestine share a geographical region, but Palestine is considered an Eastern Mediterranean country, whereas Israel is not. This makes it difficult to know whether a study’s population from this region is limited to the Eastern Mediterranean. Most studies were also carried out with medical or paramedical students, who are generally perceived as being under high academic stress. Future studies could explore the interrelatedness of suicidal ideation with medical studies among other studies of similar and dissimilar rigor, as well as identify specific interventions that could better understand and promote students’ well-being and coping in medical school. Lastly, the vast majority of studies did not control for receiving treatment for suicidal ideation, with some going to the extent of excluding participants who gained access to medication or psychotherapy [93]. Not controlling for receiving treatment could be due to the extremely limited number of students who received actual treatment for suicidal ideation, either caused by a lack mental health resources or even a lack of awareness of psychological disorders among the Middle Eastern population.

## Conclusions

Risk for suicidality among students in the eastern Mediterranean is related to demographics, individual factors, mental health, and school-related stress and bullying. Peers and family support has potential to protect students from developing suicidal ideation. Religion and cultural values play an important role in stigma and underreporting the extent of suicide in this population. The awareness of these high prevalence of suicidality among youth is low and no specific preventative measures or treatment options are reported.

## Supporting information

S1 Checklist PRISMA 2020 checklist. (DOCX)

S1 Table Newcastle-Ottawa Quality Assessment Scale. (DOCX)

S1 Appendix Search terms used. (DOCX)
