# Supplementary material for: Suicidality among university students in the Eastern Mediterranean region: A systematic review
Source: PLOS Glob Public Health. 2023 Oct 20;3(10):e0002460. doi: 10.1371/journal.pgph.0002460 (PMC10588889; doi:10.1371/journal.pgph.0002460)
Supplement: S1 Table — (DOCX) [file pgph.0002460.s002.docx]

**Quality Assessment Table**

|  |  | Modified Newcastle Ottawa Quality Assessment Scale for COHORT studies | | | | | | | | |
| --- | --- | --- | --- | --- | --- | --- | --- | --- | --- | --- |
|  |  | **SELECTION (max 4)** | | | | **COMPARABILITY (max 2)** | **OUTCOME (max 3)** | | |  |
| Author, Year | **Methodology of Study** | **Representativeness of the exposed cohort** | **Selection of the non-exposed cohort** | **Ascertainment of exposure** | **Demonstration that outcome of interest was not present at the start of the study** | **Comparability of cohorts on the basis of the design or analysis** | **Assessment of outcome** | **Was follow-up long enough for outcomes to occur?** | **Adequacy of follow up of cohorts** | **Score (/9)** |
| Norrish, M., 2009 | Within subjects, repeated-measures study | + | + | + | + | + | + | ++ |  | 8 |
|  |  |  |  |  |  |  |  |  |  |  |
|  |  | **Modified Newcastle Ottawa Quality Assessment Scale for CROSS SECTIONAL studies** | | | | | | | | |
|  |  | **SELECTION (max 5)** | | | | **COMPARABILITY (max 2)** | **OUTCOME (max 3)** | |  |  |
| Author, Year | **Methodology of Study** | **Representativeness of the sample** | **Sample size** | **Non-respondents** | **Ascertainment of the exposure** | **Comparability based on the study design or analysis** | **Assessment of the outcome** | **Statistical test** | **Score (/10)** | |
| Abdel-Khalek, A. M., & Lester, D., 2007 | Cross sectional study | + | ++ |  | ++ | ++ | ++ | + | 10 | |
| Abdel-Khalek, A., & Lester, D., 2002 | Cross-sectional study | + | ++ |  | + | + | ++ | + | 8 | |
| Abdollahi, A., & Carlbring, P., 2017 | Cross sectional questionnaire-based survey | ++ | + |  | + | ++ | + | + | 8 | |
| Ahmadboukani, S. et al., 2021 | Descriptive and modelling study | + | + |  |  | ++ | + | + | 6 | |
| Ahmadi, J. et al., 2014 | Cross sectional study | + | + |  | + | ++ | ++ | + | 8 | |
| Ahmadpoor, J. et al., 2020 | Cross sectional questionnaire-based survey | ++ | + | + | + | + | + | + | 8 | |
| Ahmed, S. A. et al., 2016 | Cross sectional study | ++ | ++ |  | + | ++ | ++ | + | 10 | |
| Akbari, A. et al., 2017 | Descriptive correlational study | + | + |  | + | + | + | + | 6 | |
| Akram, B. et al., 2018 | Cross sectional analytical study | + | + |  | + | + | + | + | 6 | |
| Almoammar, S. et al., 2021 | Cross sectional study online questionnaire-based study | ++ | ++ |  | + | + | + | + | 8 | |
| Amiri, L. et al., 2013 | Cross sectional study | + | + |  | + | ++ | ++ | + | 8 | |
| Amr, M. et al., 2013 | Cross sectional study | ++ | ++ |  | + | + | + | + | 8 | |
| Ariapooran, S., & Sheibani, H., 2021 | Descriptive and correlational study | + | + |  |  | + | + | + | 5 | |
| Bibi, A. et al., 2019 | Cross sectional study | + | ++ |  | ++ | ++ | + | + | 9 | |
| Borji, M. et al., 2019 | Descriptive-analytical study | ++ | + |  | ++ | ++ | + | + | 9 | |
| Dadfar, M. et al., 2021 | Instrument validation study | + | ++ |  | + | ++ | + | + | 8 | |
| Dar, S. et al., 2022 | Correlational study | + | + |  | ++ | ++ | + | + | 8 | |
| Elhadi, M. et al., 2020 | Cross sectional study | + | ++ |  | + | + | ++ | + | 8 | |
| Eskin, M. et al., 2020 | Cross sectional  correlational design study | ++ | ++ |  | + | ++ | + | + | 9 | |
| Eskin, M. et al., 2021 | Cross sectional questionnaire-based survey | + | ++ |  | ++ | ++ | + | + | 9 | |
| Fekih-Romdhane, F. et al., 2020 | Cross sectional study | + | ++ |  | + | + | + | + | 7 | |
| Fekih-Romdhane, F. et al., 2021 | Cross sectional study | + | ++ | + | + | + | + | + | 8 | |
| Ghadampour, E. et al., 2017 | Correlational study | + | + |  | + | + | ++ | + | 7 | |
| Ghaderi, D. et al., 2018 | Causal-comparative study | ++ | + |  | + | ++ | + | + | 8 | |
| Goldney, R. D. et al., 1998 | Cross sectional study | + |  |  | + |  |  | + | 3 | |
| Habibi, F. et al., 2021 | Cross sectional descriptive study | + | + |  | + | + | + | + | 6 | |
| Habib, O., et al., 2022 | Cross sectional descriptive study | + | + |  | + | + | + | + | 6 | |
| Hakami, R. M., 2018 | Cross sectional study | ++ | ++ |  | + | + | ++ | + | 9 | |
| Hamdan, S., & Hallaq, E., 2021 | Cross sectional questionnaire-based survey | + | + |  | + | ++ | ++ | + | 8 | |
| Heydari, A. et al., 2013 | Cross sectional questionnaire-based survey | + | ++ |  | + | + | + | + | 7 | |
| Hidarisharaf, P. et al., 2016 | Descriptive and correlational study | + | + |  | + | + | + | + | 6 | |
| Hosseini, A. et al., 2005 | Causal-comparative study | + |  |  | + | + | + | + | 5 | |
| Inam, S. B., 2007 | Cross sectional study | + | + |  | + | ++ | + | + | 7 | |
| Khaleghi, M. et al., 2021 | Cross sectional study | + | ++ |  | + | ++ | + | + | 8 | |
| Khokher, S., & Khan, M. M., 2005 | Cross sectional study | + | + |  | + | + | + | + | 6 | |
| Khosravi, M., & Kasaeiyan, R., 2020 | Cross sectional study | + | ++ |  | + | ++ | ++ | + | 9 | |
| Kiaei, Y., & Kachooei, M., 2022 | Descriptive study | + | ++ |  | + | ++ | ++ | + | 9 | |
| Klibert, J. J. et al., 2021 | Cross sectional, correlational design, cross-national study | + | ++ |  | + | + | ++ | + | 8 | |
| Landrault, H. et al., 2020 | Cross sectional study | + | + |  | + | + | ++ | + | 7 | |
| Madadin, M. et al., 2021 | Cross sectional study | + | + |  | + | + | ++ | + | 7 | |
| Modaresesabzevari, S., & Nouri Ghasemabadi, R., 2016 | Cohort study | + | + |  |  | + | + | + | 5 | |
| Mohamed, M. et al., 2023 | Cross-sectional comparative study | ++ | + |  | + | + | ++ | + | 8 | |
| Mohammadinia, N. et al., 2012 | Analytic- descriptive; cross sectional study | ++ | + |  | ++ | + | + | + | 8 | |
| Monirpoor, N. et al., 2014 | Cross sectional study | + | ++ |  | + | + | + | + | 7 | |
| Moradi, A. et al.,  2009 | Descriptive and post-event study | ++ | + |  | + | ++ | + | + | 8 | |
| Mousavi, S. G. et al.,  2005 | Descriptive-analytic, cross sectional study | + | + |  |  | + | + | + | 5 | |
| Mousavi, S. G. et al., 2012 | Cross sectional study | ++ | ++ |  | + | ++ | ++ | + | 10 | |
| Movahedi, Y. et al., 2013 | Cross sectional descriptive study | ++ | + |  | + | + | + | + | 7 | |
| Mufti, R. et al., 2022 | Cross sectional | + | + |  | + | ++ | + | + | 7 | |
| Muneeb, N. & Masood Ul Hassan, S., 2022 | Cross sectional study | ++ | + |  | ++ | + | ++ | + | 9 | |
| Naghavi, A. et al., 2020 | Cross sectional study | + | ++ |  | ++ | ++ | ++ | + | 10 | |
| Naseem, S. & Munaf, S., 2017 | Cross sectional study | ++ | ++ |  | + | ++ | ++ | + | 10 | |
| Panaghi, L. et al., 2010 | Descriptive study | ++ | + |  | + | ++ | + | + | 8 | |
| Poorolajal, J. et al., 2017 | Cross sectional study | + | ++ |  | + | + | + | + | 7 | |
| Poorolajal, J. et al., 2019 | Cross sectional questionnaire-based survey | ++ | + |  | + | + | + | + | 7 | |
| Pournaghash-Tehrani, S. S. et al., 2019 | Cross sectional study | ++ | ++ |  | + | ++ | ++ | + | 10 | |
| Poursharifi, H. et al., 2012 | Interventional research study | + | + |  | ++ | + | + | + | 7 | |
| Raeisei, A. et al., 2015 | Cross sectional study | + | + |  | + | ++ | ++ | + | 8 | |
| Rahimi, N., & Asadollahi, Z., 2016 | Cross sectional study |  | + |  | ++ | + | + | + | 6 | |
| Rashid, S. et al., 2017 | Descriptive correlation study |  | + |  | + | + | + | + | 5 | |
| Rashid, S. et al., 2020 | Descriptive correlation study | + | + |  |  | + | + | + | 5 | |
| Sadri Damirchi, E. et al., 2018 | Descriptive and correlational study | + | + |  | + | + | + | + | 6 | |
| Sadri Damirchi, E. et al., 2019 | Correlational study |  | + |  |  | + | ++ | + | 5 | |
| Salman, M. et al., 2022 | Cross sectional study | ++ | + |  | ++ | + | ++ | + | 9 | |
| Shahbaziyankhonig, A. et al., 2020 | Causal-comparative study | ++ | + |  | + | + | + | + | 7 | |
|  |  |  |  |  |  |  |  |  |  | |
| Shamsaei, F. et al., 2019 | Cross sectional questionnaire-based survey | ++ | + |  | + | + | + | + | 7 | |
| Shawahna, R. et al., 2020 | Cross sectional observational design study | + | ++ |  | ++ | + | + | + | 8 | |
| Sogoli, F., 2017 | Descriptive, cross sectional study | + | + |  | + | + | + | + | 6 | |
| Talih, F. et al., 2018 | Cross sectional study | + | ++ |  | + | ++ | ++ | + | 9 | |
| Tarsafi, M. et al., 2015 | Cross sectional study | + | + |  | + | ++ | + | + | 7 | |
| Valilkhani, A., & Firooz Abadi, A., 2015 | Descriptive and correlational study | + | + |  |  | + | + | + | 5 | |
| Zarei, M., 2021 | Descriptive and correlational study | ++ | + |  | + | + | + | + | 7 | |
| Zemestani, M., et al., 2023 | Cross-sectional | ++ | + | + | + | ++ | + | + | 9 | |
