# Supplementary material for: Suicidality among university students in the Eastern Mediterranean region: A systematic review
Source: PLOS Glob Public Health. 2023 Oct 20;3(10):e0002460. doi: 10.1371/journal.pgph.0002460 (PMC10588889; doi:10.1371/journal.pgph.0002460)
Supplement: S1 Appendix — (DOCX) [file pgph.0002460.s003.docx]

**Appendix A**

To find articles dealing with the relationship between post-secondary students and suicide in Eastern Mediterranean countries, the authors used a combination of the following search terms: [Suicidal outcomes (e.g., suicide OR suicidal OR self-directed violence OR parasiticide OR self-harm) AND population-related terms (e.g., students OR

universities OR college OR high school OR higher education OR undergraduate

OR graduate OR master OR doctoral OR bachelor OR postgraduate OR schools)

AND country-related terms (e.g., Middle East OR Africa, Northern OR Iran OR Egypt

OR Jordan OR Kuwait OR Lebanon OR Libya OR Morocco OR Occupied

Palestinian Territories OR Palestine OR Oman OR Pakistan OR Qatar OR Saudi

Arabia OR Somalia OR Sudan OR Syrian Arab Republic OR Tunisia OR United

Arab Emirates OR Yemen)].
